# Artificial Intelligence in Infectious Disease Clinical Practice: An Overview of Gaps, Opportunities, and Limitations

**DOI:** 10.3390/tropicalmed9100228

**Published:** 2024-09-30

**Authors:** Andreas Sarantopoulos, Christina Mastori Kourmpani, Atshaya Lily Yokarasa, Chiedza Makamanzi, Polyna Antoniou, Nikolaos Spernovasilis, Constantinos Tsioutis

**Affiliations:** 1 School of Medicine, European University of Cyprus, 2404 Nicosia, Cyprus; as192173@students.euc.ac.cy (A.S.); cm191479@students.euc.ac.cy (C.M.K.); arthsha24@hotmail.com (A.L.Y.); cm191347@students.euc.ac.cy (C.M.); pa192176@students.euc.ac.cy (P.A.); 2 Brigham Women’s and Children Hospital, Boston, MA 02115, USA; 3 Department of Infectious Diseases, German Oncology Centre, 4108 Limassol, Cyprus; nikolaos.spernovasilis@goc.com.cy; 4 School of Medicine, University of Crete, 71110 Heraklion, Greece

**Keywords:** artificial intelligence, clinical medicine, data privacy, deep learning, diagnosis, ethics, limitations, machine learning, personalized medicine, research, therapeutics

## Abstract

The integration of artificial intelligence (AI) in clinical medicine marks a revolutionary shift, enhancing diagnostic accuracy, therapeutic efficacy, and overall healthcare delivery. This review explores the current uses, benefits, limitations, and future applications of AI in infectious diseases, highlighting its specific applications in diagnostics, clinical decision making, and personalized medicine. The transformative potential of AI in infectious diseases is emphasized, addressing gaps in rapid and accurate disease diagnosis, surveillance, outbreak detection and management, and treatment optimization. Despite these advancements, significant limitations and challenges exist, including data privacy concerns, potential biases, and ethical dilemmas. The article underscores the need for stringent regulatory frameworks and inclusive databases to ensure equitable, ethical, and effective AI utilization in the field of clinical and laboratory infectious diseases.

## 1. Introduction

The advent of artificial intelligence (AI) has heralded a transformative era in clinical medicine, offering opportunities to enhance diagnostic speed and accuracy, optimize therapeutic efficacy, and benefit healthcare in general [1]. Driven by unprecedented technologic advancements, AI has been increasingly integrated into healthcare settings. These innovations enable AI systems to process vast amounts of data, recognize complex patterns, and make informed decisions, often with greater speed and accuracy than traditional methods [2]. Despite the profound implications of these capabilities, they are not without their limitations and challenges.

The current manuscript delves into the multifaceted applications of AI in medicine, particularly focusing on its role in addressing infectious diseases. By examining current potential and uses, possible benefits, as well as limitations and ethical challenges, we aim to provide a comprehensive overview of the impact of AI on different aspects of the diagnosis, surveillance, and management of infectious diseases. We highlight key areas where AI can fill existing gaps, offering solutions for rapid disease detection, accurate diagnostics, outbreak surveillance, management, and personalized management approaches, underscoring the critical role of AI in advancing healthcare while acknowledging the challenges that must be addressed to fully realize its potential and ensure optimal health outcomes.

## 2. Structure and Function of AI in Clinical Medicine

**Figure 1:**
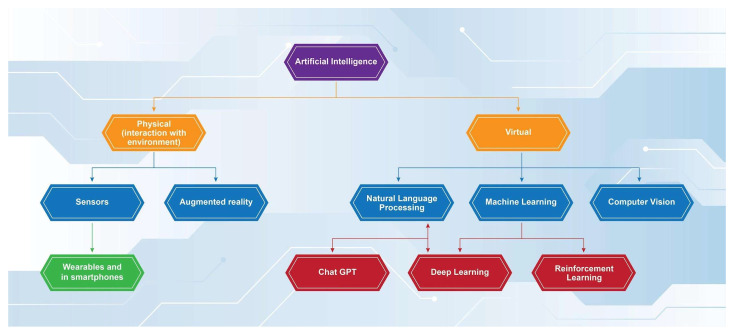
Functions of artificial intelligence.

The use of AI in medicine can be divided into two broad categories based on how it functions: virtual and physical [3]. Physical AI refers to machines that can assist in and perform different tangible tasks, such as surgical operations and the application of robotics in medical interventions. Virtual AI includes all the software that can be used to provide analysis of data, process information, and communicate with other network-connected systems (Figure 1).

More specifically, virtual AI utilizes two main techniques to compute data and provide evidence-based replies. Through machine learning (ML)—the ability of AI software to be programmed with appropriate algorithms leading to decision making and/or predictions based on the data available [2]—it can provide accurate decisions regarding the medical condition of the patient and their treatment in “real-time”. This is the so-called flowchart technique, and it mimics the way a physician gathers his information when taking the patient’s history and reviewing the results of clinical tests. In addition, AI software that uses a more advanced version of ML called deep learning (DL) can utilize the second technique, the database approach.

DL is defined as “a subtype of ML with the ability to utilize artificial intelligence networks for analyzing and processing big datasets” [4]. An important additional feature of this function is the power to recognize specific patterns of information not only from pre-labeled data but also from “raw” unprocessed data [5]. The database approach enables AI programs to accurately recognize patterns after being appropriately “trained”. Further development in the aforementioned field has led to the evolution of deep learning models into reinforcement learning models (RLs). RLs function more similarly to the way humans perform decision making and cognitive associations, since they are programmed with a focus on providing optimal results in each situation [6]. This ability can be essential in the utilization of RL AI models in the clinical setting, as they can potentially provide a coherent differential diagnosis with an associated optimal management plan for each separate patient case [6]. Different models of RL have been applied in the area of medical diagnosis, aiming to reduce diagnostic errors and optimize patient management, using automated diagnostic procedures and providing individualized treatment strategies [7]. Such examples in the relevant literature include the successful use of RL models in sepsis management, medical imaging, and HIV treatment, exhibiting better outcomes compared to traditional approaches [7,8,9].

In addition, with the development of natural language processing (NLP)—the ability of AI to understand and interpret input in human language [2]—the need for manual input from the physician is minimized, and rapid decision making from a significant amount of data can be achieved. NLPs have recently further developed into large language models (LLMs). LLMs utilize deep learning not only to comprehend human language but also to make linguistic and contextual associations of the words used, without necessarily being trained in the specific lingual task [10]. Thus, LLMs can further comprehend history taken directly from patients in a more human-like manner, while understanding not just specific words but also the meaning and context of the whole patient’s report.

## 3. AI Applications in Clinical Medicine

Artificial intelligence software—either physical or virtual—has variable applications in the clinical medicine setting. First and foremost, AI software provides invaluable tools in everyday clinical examination. Through the use of NLP, the AI software can record a coherent patient history by analyzing the voice of both the patient and the physician. In addition, it can underline points of significance in the history, such as previous treatments, comorbidities, and relevant symptoms [11,12]. AI can also be used to enhance the actual clinical evaluation. A characteristic example is the AI-augmented stethoscope. This device can recognize and interpret abnormal sounds during auscultation [13]. Other similar devices use sound waves to diagnosis Parkinson’s disease and COVID–19 [14]. Last but not least, AI programs can analyze the patient’s data and provide a list of differential diagnoses based on probability models, international guidelines, and the incorporation of textbooks on general pathology and clinical medicine.

In addition, LLM AI software can communicate directly with the patient and provide reports and proper guidance based on the history and the examination results. More specifically, AI chatbots have demonstrated the ability to effectively identify diagnostic elements in medical reports and offer potential diagnosis and treatment options, while maintaining an appropriate tone toward the patient [15,16]. Despite this, AI chatbots can still lead to misinterpretation and misdiagnosis; thus, physician overview before the report is further used or given to the patient is still a necessity [15].

Imaging and therapeutics can also utilize AI software to increase their accuracy and efficacy. More specifically, AI DL programs can be trained to recognize pathological patterns using international databases of imaging findings. The programs can provide real-time diagnosis of the findings of the test and suggest additional points of interest that the physician might have missed at first glance. The aforementioned AI algorithms have been applied in the diagnosis of tuberculosis, ischemic stroke, breast cancer, and melanoma [17,18,19,20].

Furthermore, AI can offer substantial development in the fields of laboratory diagnostics. Computational pathology is a field of laboratory pathology that utilizes deep neural network-based algorithms to provide results for histology slides, multi-omics data, and clinical informatics [2]. It is important to mention that AI software does not only analyze the morphological patterns of the input provided, but also utilizes clinical knowledge databases to make connections between findings and potential diagnoses and management strategies.

Currently, digital pathology detection has been developed by *PathAI* with the ability to analyze histopathology samples, providing diagnoses and subsequent patient-specific treatment options based on the characteristics of samples [21]. This technology has been applied to the detection of PD-L1 and HER2 proteins implicated in NSCLC and breast cancer, respectively. Moreover, this technology has the ability to characterize the specific tumor microenvironment and provide tumor grading and staging based on analysis of the severity and spread of fibrosis [21]. This has been achieved with *PathExplore*, *AIM-PDl-L1*, and *AIM-HER2* products developed by *PathAI* using ML-AI. Other products developed by *PathAI* have similar use in ulcerative colitis (UC). While *PathAI* services are currently limited to breast cancer, NSCLC, and UC, their use of ML may be applied to infectious diseases, offering prompt detection of microbial agents from patient samples [21]. Furthermore, this technology demonstrates the ability to identify possible drug-resistant features of pathogens through the application of ML to known features of resistant microorganisms, and thus may offer treatment options to bypass antimicrobial resistance.

Additionally, AI-powered imaging analytics have been developed by *Aidoc* with the ability to interpret imaging studies including X-rays, echocardiograms and CT scans using its artificial intelligence operating system (*Aidoc*) software [22,23]. This software has the ability to interpret, diagnose, assess risk, and identify any structural abnormalities in cardiovascular and neurologic imaging studies. These include cardiovascular imaging related to venous thromboembolism (VTE), coronary artery disease, and aortic syndromes; in neurologic conditions, imaging related to acute trauma and neurovascular and spinal conditions can be interpreted using the *Aidoc* software v.1.3 [22,23]. More specifically, in cardiovascular care, for example, the software uses a *PE* and *iPE algorithm* to detect suspected and incidental pulmonary emboli, respectively, and can detect pulmonary arterial hypertension and risk of death or deterioration by calculating the right ventricle to left ventricle ratio using an *RV/LV algorithm* from CTPA. Various algorithms have been developed by *Aidoc*, 17 of which have current FDA-approval for neurology and cardiovascular diagnostics, including the *PE* and *iPE algorithms* for pulmonary embolism and the large vessel occlusion (LVO) algorithm used for analysis and detection of strokes in CTA scans [22,23]. Similar to the *PathAI* products, the algorithms developed by *Aidoc* have not been applied to infectious disease as yet; however, they have demonstrated the ability to be integrated in the field of radiology related to infectious disease in the near future.

It is worth noting that the application of AI in physical tasks (such as surgical operations or arterial/venous line placement) is revolutionizing the field of medicine. The use of AI in robotic surgery allows the real-time enhancement of images and therefore easier identification of anatomical structures. Ali et al. have created an online preprocessing framework designed to enhance real-time camera imaging in knee arthroscopy by denoising, deblurring, and correcting colors for better intraoperative visualization, outperforming any existing image enhancement methods [24]. Moreover, Wang et al. have developed a method using a convolutional neural network (CNN) paired with a Swim transformer to remove smoke from surgical footage, resulting in a clearer view during operations and emergency situations such as acute intraoperative hemorrhage. Finally, AI anatomic mapping software, such as the FDA-approved Cydar EV Maps (Cydar Medical, Cambridge, UK), is steadily gaining ground in everyday clinical practice for interventional endovascular procedures, providing a significant reduction in patient radiation exposure and overall operative safety improvements [25].

## 4. Filling the Gaps in Clinical Infectious Diseases—How AI Can Contribute

**Figure 2:**
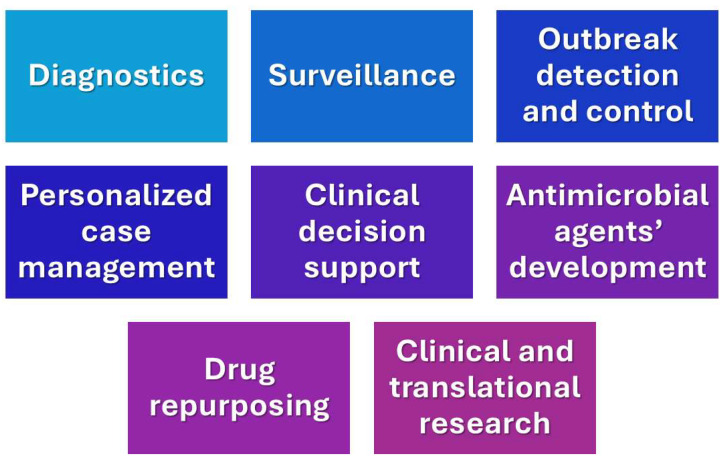
Current applications of AI in clinical infectious diseases.

Infectious diseases continue to pose significant challenges to global public health systems, thus revealing various gaps in diagnostics, rapid disease detection, treatment optimization and therapeutic algorithms, outbreak detection and management, and generally, in personalized medicine. In the 21st century, various infections have emerged, leading to pandemics, including severe acute respiratory distress syndrome (SARS), Ebola, Middle East respiratory syndrome (MERS), and Zika viruses [26]. These infectious diseases are responsible for a great number of premature deaths and increasing the burden of disability worldwide. Infectious diseases caused by transmissible pathogens pose a challenge for clinicians, despite advances in medicine, due to the expanding prevalence of antimicrobial resistance and limitations to the fast and accurate detection of these microorganisms. Biological complexity also poses a great obstacle in understanding and dealing with infectious diseases. AI can play a significant role in extending our knowledge and facilitating our efforts in various areas of infectious diseases [27], including laboratory diagnostics, clinical imaging, clinical decision making, surveillance, management outbreaks, and drug discovery (Figure 2).

### 4.1. Diagnostics

In recent years, AI has been one of the most efficient tools in diagnosing different diseases either through laboratory-based or medical imaging diagnostics. Precisely, laboratory-based diagnostics are usually limited in resource-constrained settings, leading to delays in diagnosis. In contrast, AI-based diagnostic tools are portable and cost-effective due to their ability to analyze laboratory samples efficiently and accurately [28]. AI is a pivotal tool in modern radiology as it provides improved diagnostic accuracy, workflow efficiency, and personalized patient care [29]. These advancements vary from image partitioning, categorization, computer-aided diagnosis, and innovative diagnostic and prognostic tools intending to improve the patient’s outcome [29].

Specifically, ML and DL models are utilized for the early detection of various diseases using non-invasive imaging models. The application of AI in the diagnosis of pulmonary tuberculosis has been achieved using computer-assisted analysis of chest radiographs and computed tomography. There was a sensitivity of 91% and specificity of 65% in a meta-analysis of 23 clinical studies with a total of 124,959 patients [30]. However, certain limitations arise, including study design heterogeneity, sample size, and the lack of independent clinical application. Some of the techniques that are currently in use for rapid diagnosis are the Boltzmann machine, K nearest neighbor, support vector machine, decision tree, logistic regression, fuzzy logic, and artificial neural networks [12]. Undoubtedly, the diagnosis of an infectious disease using traditional diagnostic methods has shown limitations in accuracy, speed, and the accessibility of such tests. On the contrary, using AI techniques, patient data are added to the analysis, thus allowing for a more rapid and accurate diagnosis. These AI-based diagnostic algorithms are continuously updated with new data and therefore improve over time [30].

Computer-assisted analysis of blood films has also been used in the diagnosis of malaria with a sensitivity of over 99% shown by some models. In a study published by Go et al., red blood cells (RBCs) infected with malaria were detected by machine learning algorithms using digital in-line holographic microscopy data [31]. Individual RBC holograms were tagged with various parameters, and statistical significance between healthy and infected RBCs was observed in 10 of them. The machine learning algorithms were applied to increase the diagnostic accuracy, and the model trained by the SVM showed the best accuracy in separating healthy from infected RBCs for training (*n* = 280, 96.78%) and testing sets (*n* = 120, 97.50%).

Another application of AI can also be seen in the detection of antimicrobial resistant organisms when AI is combined with conventional methods of diagnosis, such as whole genome sequencing and matrix-assisted laser desorption/ionization time of flight mass spectrometry (MALDI-TOF). The increasing antimicrobial resistance poses a threat to the effective prevention and treatment of an ever-increasing range of bacterial, viral, fungal, and parasitic infections. In a study that was published by Weis et al., the researchers developed a new way to predicting antimicrobial resistance by combining ML with MALDI-TOF using a database of mass spectra profiles from the clinically most relevant isolates with linked antimicrobial susceptibility phenotypes [32]. Around 300,000 mass spectra with 750,000 antimicrobial resistant phenotypes were combined from four different medical institutions. With this new approach, resistant pathogens including *Staphylococcus aureus*, *Escherichia coli*, and *Klebsiella pneumoniae* were detected, demonstrating the possibility of ML as a helpful tool to considerably accelerate antimicrobial resistance determination and alteration of clinical management. Lastly, a retrospective clinical case study that included 63 patients indicated that the clinical treatment would have been altered in nine cases if this new approach was implemented and eight of these cases (89%) would have benefited.

Apart from laboratory diagnosis, AI can also be used to support clinical decision making in the context of prediction and stratification of sepsis and antimicrobial stewardship advice. Several studies were published researching the real-world implementation of machine learning algorithms (MLAs) to aid in the early detection of sepsis. Shimabukuro et al. conducted a randomized controlled trial involving adult patients in two intensive care units (ICUs) at the University of California, San Francisco Medical Center [33]. The average length of stay and in-hospital mortality were evaluated. The MLA was used in the experimental group, whereas the current severe sepsis detector was used in the control group. The outcome was a statistically significant reduction in the MLA group, in comparison to the control group, in both length of hospital stay and in-hospital mortality. More specifically, average length of stay decreased from 13.0 days in the control to 10.3 days in the experimental group, and in-hospital mortality decreased by 12.4 percentage points. These results highlight the importance of AI as a tool in the early detection of infection and therefore better clinical outcomes.

### 4.2. Surveillance and Outbreak Detection

AI is also a useful tool in case analysis by collecting and analyzing information on disease transmission patterns, risk factors, and clinical outcomes. Utilizing this, healthcare professionals could identify high-risk populations, trace the spread of diseases, and predict potential outbreaks. This tends to enhance early intervention strategies while optimizing the utilization of resources [34].

Therefore, outbreak detection and management are of utmost importance, as highlighted throughout history. Traditional surveillance methods have to perform data collection manually, which in the long run causes delays in reporting or under-reporting infectious disease cases. In contrast, AI-based surveillance systems automatically collect data both from hospital records and social media while analyzing real-time data streams to increase situational awareness and enhance the public health response [35]. Finally, AI-based warning systems have been created that detect irregularities in the incidence of a disease or pattern of infectious disease transmission and then inform healthcare authorities of potential outbreaks [36].

### 4.3. Personalized Medicine

In addition to the above current uses of AI in medicine, its presence in personalized medicine is key. AI analyses patient data, including genetic makeup and treatment history, to create a personalized and optimized treatment for the patient. Alternatively, personalized healthcare monitoring systems using Bluetooth-based sensors and real-time data processing are used to collect the patient’s vital signs and report them back on a smartphone for disease monitoring. Nonetheless, these systems are also used to support healthcare professionals in treating patients by assisting them in the selection of the appropriate treatment regimen [12].

Additionally, a study published by Paul et al. reviewed the efficacy of an AI therapeutic algorithm system known as the TREAT decision-support system that aids in optimizing antibiotic therapy, while reducing any unnecessary treatment for patients [37]. However, this is only one of many attempts to use AI to optimize treatment, and supervised ML is used to tailor the treatment plan of patients to optimize their therapeutic outcomes [38].

Recent studies have also shown the potential for AI in vaccine development, especially in the development of mRNA vaccines using ML [39]. These studies, discussed further in the current review, illustrate how AI can be used to optimize vaccination innovation—that is, to ensure the highest possible efficacy of mRNA vaccines are created. Specifically, AI can be used to maximize stability, ensure a prolonged half-life of mRNA vaccines, reduce reliance on expensive cold-chain technology currently required for storage, and significantly reduce the production time of vaccines [40].

The above-mentioned uses of AI in mRNA vaccines production were widely highlighted in the recent COVID-19 pandemic. mRNA vaccines are being used to limit the spread of COVID-19 infection; however, certain limitations arise, including the instability and degradation of mRNA molecules, which results in the reduction of the efficacy of the vaccine products and compromised immunogenicity. In a study published by Zhang et al., the goal was to develop an mRNA molecule that is characterized by increased stability, greater potency, and promising clinical efficacy [40]. Limitations in achieving the desirable mRNA design include the exponentially large search space. Each amino acid is encoded by a triplet codon; however, most amino acids have multiple codons, resulting in a wide range of candidates for any protein sequence. The development of an algorithm called LinearDesign, which includes the concept of lattice parsing in computational logistics, addressed this limitation. This algorithm was used to drastically improve mRNA half-life and protein expression and increase the antibody titer by up to 128 times compared to the codon optimization benchmark for mRNA vaccines against COVID-19 and varicella-zoster virus. This algorithm showcased that discovering the optimal mRNA among various candidates is analogous to finding the most probable sentence among many similar-sounding alternatives. This seems to be a valuable tool in not only COVID-19 vaccines but also other mRNA-based medicines encoding therapeutic proteins, such as monoclonal antibodies and anti-cancer drugs. The use of LinearDesign resulted in substantial improvements in chemical stability in vitro, protein expression in cells, and the in vivo immunogenicity of COVID-19 and varicella-zoster virus (VZV) mRNA vaccines.

AI is also an important tool in drug discovery and infectious disease research. Recently, Stokes et al. published a study where the novel antimicrobial halicin was identified from a screen of over 6000 compounds in the Drug Repurposing Hub database using a deep neural network. Halicin is structurally different from other antibiotics, showed broad-spectrum bactericidal activity against *Mycobacterium tuberculosis* and carbapenem-resistant Enterobacteriaceae, and effectively treated *Clostridioides difficile* and *Acinetobacter baumannii* infections. This study emphasizes the utility of DL approaches to discover antibacterial molecules with unique structures and consequently combat the rapid increase in antimicrobial resistance [41]. Interestingly, AI tools can be used in research to serve different functions. For example, a systematic review assessing IT-related initiatives during the first wave of the COVID-19 pandemic in Italy employed NLP techniques to analyze the textual contents of data retrieval, and then ML models were used to group these findings into clusters [42].

## 5. Limitations in the Use of AI

Although AI can benefit and support the field of infectious diseases, some limitations have been reported.

The use of AI modalities, such as ChatGPT, entails potential issues, such as disease misinformation, lack of human interaction, accessibility, and language barriers [43]. ChatGPT can cause misinformation as it is trained on input data. If the input data contain misinformation, then the responses are inaccurate. On another hand, the lack of human interaction is evident and can play a huge role as patients need emotional support and a detailed, clear explanation of the condition. Accessibility is another issue when using AI, such as the lack of internet access for patients and medical professionals, especially in regions with limited connectivity. The language barrier is another limitation when using certain AI tools as they are available in only a few languages, which could pose a challenge for both patients and physicians [43].

Additionally, using AI in imaging has been demonstrated to be a valuable asset for physicians, but numerous scientific limitations are currently being faced. These include a lack of disease specificity, data scarcity, and spectrum bias. Due to the AI models being trained only on in vivo imaging modalities, there is a limitation in their potential performance as biomarkers of infectious disease progression [44]. Nonetheless, there are currently some PET tracers that are being designed to be specific for bacteria; however, they are still utilized in a preclinical setting. Moreover, data scarcity presents another limitation for AI in imaging, as these models require a large number of labelled samples for adequate training. Specifically, some diseases tend to have more available human data, while other diseases tend to have more data from animal models. Furthermore, using AI models in a clinical setting has raised concerns of spectrum bias as this could affect the performance of the AI models based on factors such as sex, age, or ethnic minorities. In preclinical research, animal demographics are more rigorously controlled due to the differences in disease presentation across various species, which leads to spectrum bias [44].

In research, significant bias can be introduced by the utilization of AI. In more detail, if the research question is formulated by the AI software, there could be potential selection bias, such as a gender bias or racial bias, that occurs since specific population sub-groups may be “favored” by the algorithm based on disease prevalence and the demographic data utilized [45,46]. In addition, AI software cannot identify if the cohorts included in its research methodology are representative of the population, leading to both sampling and classification bias [45]. Last but not least, AI research software can lead to a phenomenon called “overfitting”. When “overfitting” occurs, the AI model showcases high performance when applied to its dataset but cannot reproduce similar results when the application is generalized to different datasets [45].

## 6. Legal and Ethical Issues in the Use of AI

There are several ethical and legal challenges regarding use of AI in infectious diseases. Health equity, social justice, patient autonomy, data protection, and privacy are some of the main areas of concern. AI algorithms require an extensive amount of personal health data, raising worries regarding privacy and the confidentiality of critical medical information. The health data can be shared with third party data aggregators, after being anonymized. When this is done, there is still a risk of re-identification through new data linkage methods. This was shown in a 2018 study with analyzed data from the National Health and Nutrition Examination [47]. In this study, it was demonstrated that an algorithm was able to re-identify a significant percentage of children and adults in a cohort study, despite being anonymized. There are also concerns about sharing data between jurisdictions due to varying laws depending on where the personal data are generated, processed, and utilized for deep learning algorithms. In Europe, health-related personal data are safeguarded under the European Union’s General Data Protection regulation. In the United States, there are more health-specific laws, such as HIPAA. These differences put the data in a more vulnerable position, with a higher risk of exploitation [47].

The General Data Protection Regulation (GDPR) focuses on the protection of people’s personal data in the European Union [48]. The GDPR aims to process, collect, and store personal information with the idea that everyone can control their own information. Key provisions include data subject rights (such as access, correction, and deletion), the requirement for companies to obtain consent, and strict penalties for non-compliance. Nonetheless, the GDPR impacts AI systems as they rely on information datasets, and the GDPR ensures that such systems comply with principles such as data minimization, transparency, and user consent [48]. In case of non-compliance with the GDPR, then penalties are implemented, which makes data protection integral to AI development and use.

Additionally, the Medical Device Regulation (MDR) is an EU law that considers AI-based healthcare tools (like diagnostic software) as medical devices [49]. However, the MDR imposes strict standards on the design, performance, and risk management of these devices with the aim to ensure the safety and efficacy of each patient [49]. Each AI-based medical devices is subject to a conformity assessment to ensure that all the requirements are met and that any risks related to software updates, bias, and reliability are reduced. Furthermore, AI-driven medical devices are classified as “high risk” due to the potential impact that they may have on the safety of the patients and thus are subjected to both the MDR and specific AI-related regulations under the AI Act, creating a dual regulatory framework.

The Health Technology Assessment Regulation (HTAR) is extremely important as it establishes a standardized process for evaluating the clinical benefits and risks of new health technologies [50]. The HTAR is usually applied when AI-based medical technologies, such as diagnostic software and robotic surgery tools, need to demonstrate their effectiveness, safety, and potential healthcare impact. It should be noted that the medical devices classified as based on the MDR and AI Act should undergo joint clinical assessments under the HTAR. It streamlines the evaluation process for AI-based health technologies, thus reducing any duplication of efforts, while ensuring that they meet the standards of the healthcare systems before any of them are adopted.

Health equity is a factor that should strongly be taken into consideration in AI development and use. Marginalized groups and minorities may be underrepresented in big health data due to lack of affordability or insurance. This can result in severe consequences when bias data and algorithms are used in real-world applications. If AI is adopted without taking these biases into account, it has the potential to exacerbate existing disparities. More inclusive databases are necessary to represent marginalized groups and minorities [51]. The COVID-19 pandemic brought more awareness to the health inequities caused by AI biases, resulting in severe consequences. AI can be used to identify and group risky patients and to provide emergency hospitalizations. Unfortunately, there have been cases where this algorithm has discriminated against minority patients. A recent scoping literature review showed significant bias across many studies during the COVID-19 pandemic, especially in the context of race and gender [52]. More specifically, concerning the U.S. healthcare system during the COVID-19 pandemic, the same level of risk was assigned to black patients as white patients, even though the black patients were sicker, resulting in insufficient hospitalization and medical care for black patients [52]. The COVID-19 mortality rate among the African-American and Hispanic communities were 3 times higher compared to the white communities [53].

The above mentioned raises the question of who will be liable for the consequences of the mistakes and errors made due to AI, bringing attention to the legal concerns regarding the use of AI. In most cases the physician is considered to be the one at fault, when it comes to malpractice. The new dilemma is if physicians can be held accountable for errors caused by AI or not [54].

On 28 September 2022, the European Commission released a proposal for an AI Liability Directive (AILD), which aims to address the concerns created by specific uses of AI by establishing regulations that prioritize safety and the upholding of fundamental rights [55]. The commission suggests adding to and updating the EU liability framework in order to include new regulations that are specific to damages brought on by AI systems. The new regulations aim to guarantee that anyone harmed by AI systems in the EU receive the same protections as individuals harmed by other technologies [55].

On 8 December 2023, the European Commission passed the world’s first comprehensive AI law called the EU AI Act. The EU AI Act was passed by the EU parliament on 13 March 2024, and will soon enter into force [56]. This regulation addresses several challenges, including racial and gender bias in AI [57]. The EU AI Act categorizes four levels of risk for AI systems. These are high, limited, unacceptable, and minimal risk. Medical devices fall into the “high risk” category. This means that the providers need to meet rigorous standards for data quality, risk management, and transparency. The manufacturers have to ensure that their products are robust against external cyberattacks in the region of the AI-based system and that any faults that may arise should be identified and regulated immediately [58].

The new regulatory rules for “high-risk” AI systems will help to minimize bias and existing systemic discrimination. It is also required that “high-risk” systems are adequately developed and tested with relevant datasets to reduce the potential incorporation of unfair biases into the model [57].

## 7. Conclusions

The integration of AI in clinical medicine, particularly in the field of infectious diseases, marks a significant advancement in healthcare, offering the opportunity to augment our capabilities in diagnostics, personalized treatment, surveillance, and therapeutic innovations. AI applications in laboratory diagnostics, medical imaging, and personalized medicine have shown potential in enhancing patient outcomes and optimizing healthcare delivery. However, the implementation of AI also brings several challenges, including technical limitations, ethical concerns, and potential biases. The lack of disease specificity, data scarcity, and the risk of spectrum bias in AI models underscore the need for continuous refinement and regulation. Ethical issues related to patient privacy, data protection, and health equity necessitate careful consideration to prevent exacerbating existing disparities. The legal implications of AI-related errors and liability also require clear guidelines and frameworks. Despite these challenges, the transformative potential of AI in clinical medicine is undeniable. As the technology evolves, it will be crucial for healthcare professionals, policymakers, and researchers to address these limitations and ethical issues. By doing so, the full benefits of AI can be realized, ensuring it serves as a valuable tool in advancing global health and improving health outcomes.

## Data Availability

No new data were created for preparation of this manuscript.
